# Insights into soybean transcriptome reconfiguration under hypoxic stress: Functional, regulatory, structural, and compositional characterization

**DOI:** 10.1371/journal.pone.0187920

**Published:** 2017-11-16

**Authors:** Thiago J. Nakayama, Fabiana A. Rodrigues, Norman Neumaier, Juliana Marcolino-Gomes, Hugo B. C. Molinari, Thaís R. Santiago, Eduardo F. Formighieri, Marcos F. Basso, José R. B. Farias, Beatriz M. Emygdio, Ana C. B. de Oliveira, Ângela D. Campos, Aluízio Borém, Frank G. Harmon, Liliane M. Mertz-Henning, Alexandre L. Nepomuceno

**Affiliations:** 1 Departamento de Fitotecnia, Universidade Federal de Viçosa, Viçosa, Minas Gerais, Brazil; 2 Embrapa Soja, Empresa Brasileira de Pesquisa Agropecuária, Londrina, Paraná, Brazil; 3 Embrapa Agroenergia, Empresa Brasileira de Pesquisa Agropecuária, Brasília, Distrito Federal, Brazil; 4 Embrapa Clima Temperado, Empresa Brasileira de Pesquisa Agropecuária, Pelotas, Rio Grande do Sul, Brazil; 5 Department of Plant and Microbial Biology, University of California-Berkeley, Berkeley, California, United States of America; Tulane University Health Sciences Center, UNITED STATES

## Abstract

Soybean (*Glycine max*) is one of the major crops worldwide and flooding stress affects the production and expansion of cultivated areas. Oxygen is essential for mitochondrial aerobic respiration to supply the energy demand of plant cells. Because oxygen diffusion in water is 10,000 times lower than in air, partial (hypoxic) or total (anoxic) oxygen deficiency is important component of flooding. Even when oxygen is externally available, oxygen deficiency frequently occurs in bulky, dense or metabolically active tissues such as phloem, meristems, seeds, and fruits. In this study, we analyzed conserved and divergent root transcriptional responses between flood-tolerant Embrapa 45 and flood-sensitive BR 4 soybean cultivars under hypoxic stress conditions with RNA-seq. To understand how soybean genes evolve and respond to hypoxia, stable and differentially expressed genes were characterized structurally and compositionally comparing its mechanistic relationship. Between cultivars, Embrapa 45 showed less up- and more down-regulated genes, and stronger induction of phosphoglucomutase (Glyma05g34790), unknown protein related to N-terminal protein myristoylation (Glyma06g03430), protein suppressor of phyA-105 (Glyma06g37080), and fibrillin (Glyma10g32620). RNA-seq and qRT-PCR analysis of non-symbiotic hemoglobin (Glyma11g12980) indicated divergence in gene structure between cultivars. Transcriptional changes for genes in amino acids and derivative metabolic process suggest involvement of amino acids metabolism in tRNA modifications, translation accuracy/efficiency, and endoplasmic reticulum stress in both cultivars under hypoxia. Gene groups differed in promoter TATA box, ABREs (ABA-responsive elements), and CRT/DREs (C-repeat/dehydration-responsive elements) frequency. Gene groups also differed in structure, composition, and codon usage, indicating biological significances. Additional data suggests that *cis*-acting ABRE elements can mediate gene expression independent of ABA in soybean roots under hypoxia.

## Introduction

Regimes of excess water (flooding) influence the distribution and diversity of species in natural ecosystems [1] and lead to yield losses of many farmland crops [2]. The increase in flooding events over the past six decades is associated with climate change, which threatens food security of the growing human population [3]. Among the four major crops (soybean, wheat, maize, and rice), only rice is adapted to soil waterlogging and all are sensitive to submergence [2].

The oxygen diffusion in water is 10,000 times lower than in air [4]. Thus, water surrounding roots (waterlogging) or entire plants (submergence) can cause severe energy crisis once oxygen is required for energy production through mitochondrial respiration [5]. Additionally, even when oxygen is externally available, oxygen deficiency is frequent metabolic status of bulky, dense or metabolically active tissues such as phloem, meristems, seeds, and fruits [6]. It is required in several metabolic pathways, including heme, sterol, and fatty-acid biosynthesis [6].

Orchestrated by complex gene regulatory network, plants and other organisms need to perceive, signal, and promote biochemical, physiological, and morpho-anatomical changes appropriate to survive and thrive under oxygen level fluctuations. The adaptation capacities of crops validated under stress field conditions have shown association with gene duplication events [7]. As an example, the multigenic SNORKEL (SK) and SUBMERGENCE-1 (SUB1) loci are found in deep-water and submergence-tolerant rice varieties, respectively [8,9]. Both loci are members of AP2 (APETALA2)/ERF (Ethylene Responsive Factor) plant-specific transcription factors superfamily. They encode tandem-repeated genes, of which SK2 [8] and SUB1A-1 [9] are majorly responsible for the rapid-growth avoidance escape (SK2) and energy saving quiescence (SUB1A-1) strategies against oxygen deprivation.

Unlike rice, soybean genome does not contain SUB1 and SK2 orthologs [10]. So far, only QTLs (Quantitative Trait Loci) with small effect for waterlogging tolerance have already been predicted in soybean [11,12]. In addition, few soybean studies of transcriptome-wide responses to flooding stress have already been reported, and those that have all examine a single genotype. Chen et al. [13] evaluated the leaf transcriptome from adult soybean seven days after waterlogging. Yin et al. [14] performed transcriptomic analysis of flooding-tolerant line and ABA-treated newly germinated seedlings under hypoxic stress. Others studies analyzed transcriptomes from shoots (cotyledons including hypocotyls) [10] and roots including hypocotyls [15,16] of newly germinated soybean seedlings under hypoxic stress.

In the present study, we analyzed conserved and divergent root transcriptional responses between flood-tolerant Embrapa 45 and flood-sensitive BR 4 soybean cultivars under hypoxic stress by using RNA-seq platform. For progress to understand how soybean genes evolve and respond to hypoxic stress, stable (SGs) and differentially expressed genes (DEGs) were structurally and compositionally characterized, comparing its mechanistic relationship with expression regulation.

## Materials and methods

### Grain yield

Under field conditions, Embrapa 45 and BR 4 seeds were sown on December 21sh, 2011, at Embrapa Clima Temperado, Pelotas, RS, Brazil (latitude 31°42'S and longitude 52°24'W). The experiment was carried out in randomized blocks, with four replicates (75 plants per plot, at a density of 200,000 plants/ha). The seed emergence occurred six days after sowing. The first waterlogging occurred 18 days after sowing due to heavy rain, lasting five days. We observed mild symptoms of yellowing leaves. On February 15th, 2012, we waterlogged the soil beds, maintaining water level 2 cm above the soil surface for 10 days. The plants developed typical reaction to waterlogging. Harvest was done on May 23th, 2012. All seeds were collected and corrected to 13% moisture content. Data met assumptions of the analysis of variance (ANOVA). Thus, means were compared by the Tukey test 5%.

### Plant material for RNA-seq and qRT-PCR analysis

Previously described [17], using a hydroponic system under greenhouse conditions, the experiment was set in a randomized block design composed of twelve treatments: two cultivars (Embrapa 45 and BR 4), two oxygen conditions [fully aerobic state (normoxy) and hypoxic], and three treatment sampling times (0.5h, 4h, and 28h). Each treatment has three biological replicates (four plantlets per replicate in order to reduce biological variation). At each time point, root tissues were collected and immediately frozen in liquid nitrogen before being stored at -80°C. We compared stressed and unstressed samples at the same time point to remove putative additive effects, such as gene-intrinsic effects (e.g., circadian rhythm [18]), differences in developmental stages among individuals, or any unknown variation between the time points [17].

Total RNA was extracted using Trizol reagent (Invitrogen) and treated with DNase I (Invitrogen) according to manufacturer instructions. RNA concentration and purity were measured using a spectrophotometer (NanoDrop, ND-1000), and the integrity of the molecules was analyzed on 1% agarose gels stained with ethidium bromide.

### Transcriptome library construction, deep sequencing, and mapping of reads

For each of twelve treatments, equimolar quantities of purified total RNA from roots of twelve plants were pooled to result one library. Then, the twelve libraries were sent to Fasteris SA (Plan-les-Ouates, Switzerland) for processing and sequencing.

The RNA quality and integrity was checked using an Agilent 2100 Bioanalyzer (Agilent, Palo Alto, CA), of which only samples with a RIN ≥ 8.0 were used. The twelve libraries were processed (poly-A purification, fractionation, cDNA synthesis using random primers, and ligation to bar-coded adapters), fragments of 150–250 bp were isolated and multiplexed, resulting one sequencing library. The sequencing library is a pool of equimolar quantities from twelve initial libraries, each library with a specific barcode for further bioinformatic discrimination. Single end reads were generated by the Illumina HiSeq 2000 (read length 1 × 100 base; one lane of the flow-cell; Illumina, Inc. San Diego, CA). The raw data, deposited in the ArrayExpress database (http://www.ebi.ac.uk/arrayexpress) under accession number E-MTAB-5709, was uploaded to the GeneSifter platform (Geospiza, Seattle, WA) for alignment with the soybean genome (assembly Glyma 1.1) [19]. The mapping of reads and transcripts analysis was done as described previously [18].

For structural analysis of non-symbiotic hemoglobin Glyma11g12980.1 transcript, reads from each cultivar were *de novo* assembled with Trinity [20] (standard parameters with minimum contig length of 400bp) and mapped to the Glyma11g12980.1 reference (Phytozome transcript model) using BWA-MEM (v0.7.5) [21] default settings.

### Transcriptomic analysis

For each time point (0.5, 4, and 28h), the expression ratio (fold-change, fc) of genes was performed by dividing transcript abundance values (in RPM = Reads per Mapped Million) from plants under hypoxic and normoxic conditions. The statistical significance of DEGs were obtained by using Bioconductor package edgeR [22], corrected by Benjamini and Hochberg method [23]. We only considered as DEGs those showing fold-change ≥ 2 (up), ≤ -2 (down), adj. *p*-value ≤ 0.01, and with more than 20 mapped reads (RPM ≥ 9) in at least one of the two compared libraries.

Gene set enrichment analysis was performed using Singular Enrichment Analysis (SEA) provided by agriGO (http://bioinfo.cau.edu.cn/agriGO/) [24]. We chose hypergeometric test, corrected by Hochberg FDR method, plant GO slim database. Soybean pathways of DEGs related to amino acids and its derivatives were analyzed using KEGG Mapper—Search&Color Pathway [25]. The Relative Synonymous Codon Usage (RSCU) was calculated with CodonW 1.4.4 (http://mobyle.pasteur.fr/cgi-bin/portal.py?#forms::CodonW). The copy numbers of soybean nuclear tRNA genes was extracted from the genomic tRNA database (http://gtrnadb.ucsc.edu/) [26]. Samples sizes used for structural and compositional analysis among groups of genes are shown in the Table A in S1 Supporting Information.

The Microsoft Excel (Microsoft, Redmond, WA) was used for TATA-box searching in promoter regions and structural/compositional characterization of genes. For hypothesis testing on binary data, we used Microsoft Excel add-in Real Statistics Resource Pack (ver. 3.3.1, http://www.real-statistics.com/).

### qRT-PCR analysis

For quantitative real-time PCR (qRT-PCR) analysis, the synthesis of cDNA, design of primers, and expression analysis of genes used to verify reliability of RNA-seq expression data were done as described previously [17] (Table D in S1 Supporting Information).

## Results and discussion

**Fig 1:**
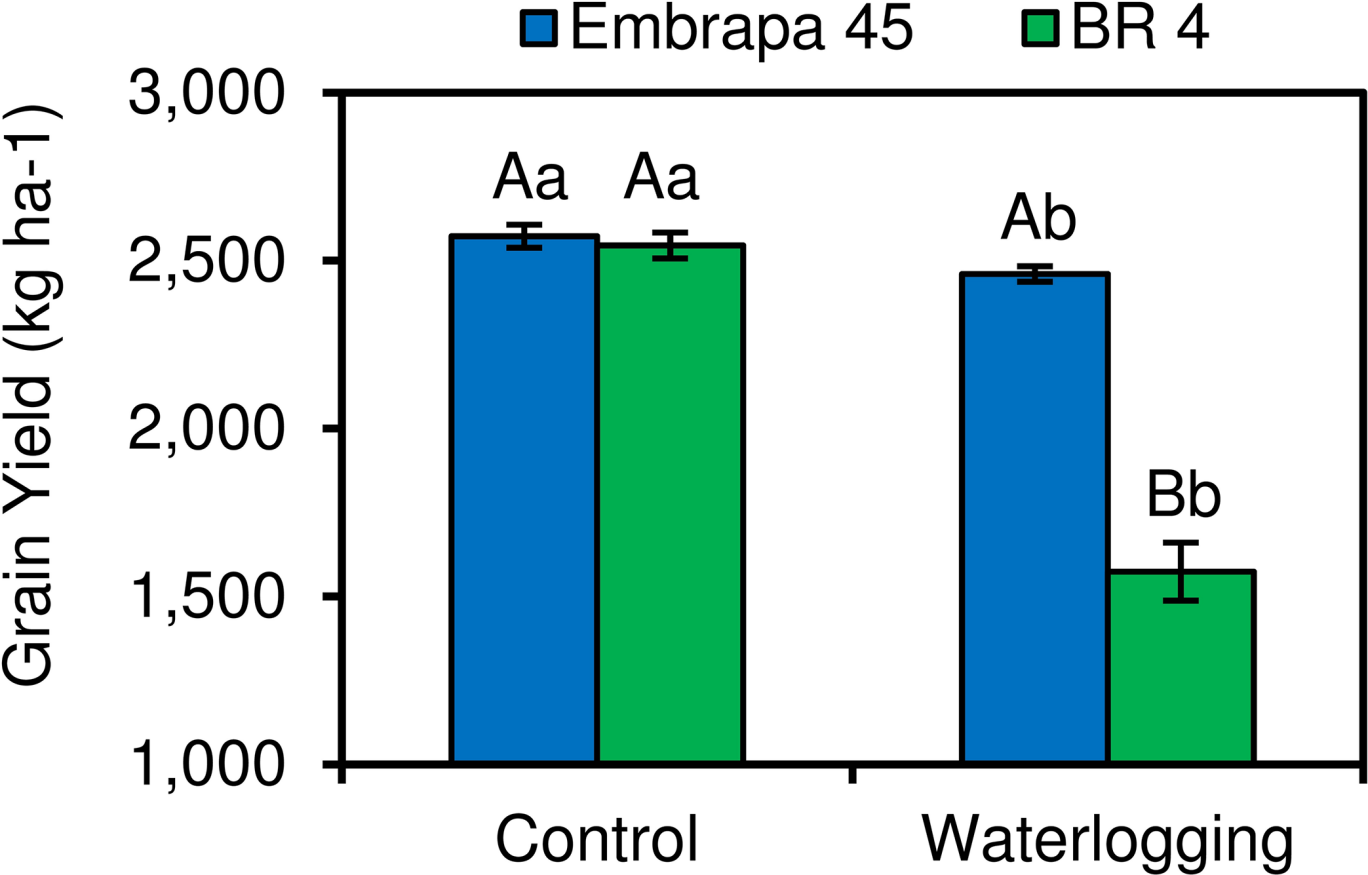
Grain yields of two soybean cultivars (flood-tolerant Embrapa 45 and flood-sensitive BR 4) under two moisture regimes in the soil. Control indicates well-watered conditions (70% available water in the soil). n = 4, except for BR 4 under waterlogging (n = 3). Each biological repetition consisted of 75 plants, from which grain yield were converted to 200,000 plants per hectare. Means values (± S.E.M.) followed by different capital letters between cultivars under same soil condition, and lowercase letters between soil conditions for same cultivar, significantly differ according to Tukey test 5%.

**Fig 2:**
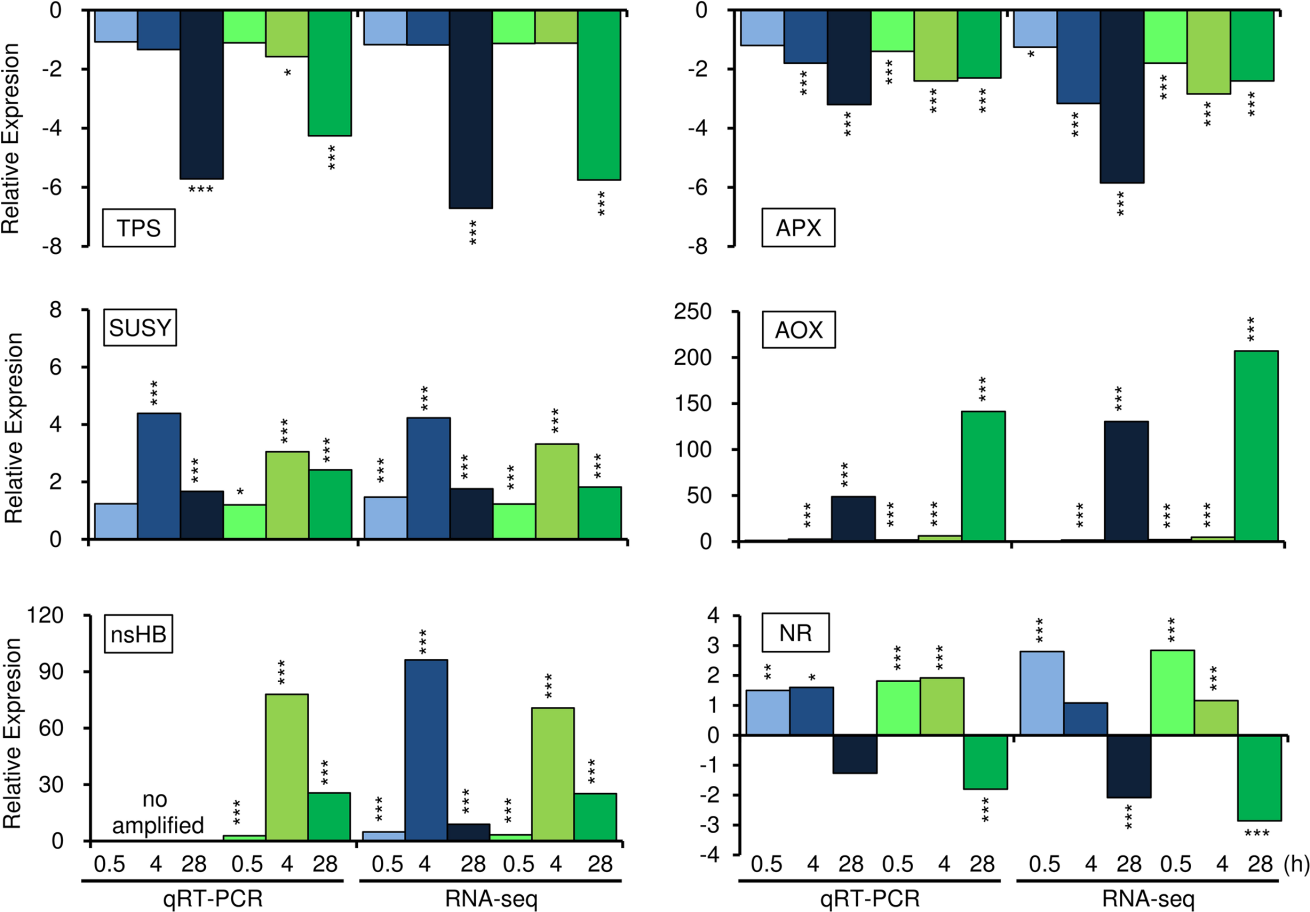
Validation of pairwise RNA-seq data through qRT-PCR. Six hypoxia-responsive genes (TPS: Trehalose-6-Phosphate Synthase, Glyma17g07530; APX: Ascorbate Peroxidase, Glyma12g07780; SUSY: Sucrose Synthase, Glyma13g17420; AOX: Alternative Oxidase, Glyma04g14800; nsHB: non-symbiotic Hemoglobin, Glyma11g12980; and NR: Nitrate Reductase, Glyma06g11430) were analyzed in flood-tolerant Embrapa 45 (Blue chart) and flood-sensitive BR 4 (Green chart) soybean cultivars. The transcripts abundance of the target genes from plants subjected to hypoxic conditions for different periods of time were compared with the respective controls (normoxic condition). Differential gene expression statistically significant: *p < 0.05, **p < 0.01, and ***p < 0.001. From qRT-PCR, raw data was normalized using the ELF1B and ACTB reference genes [17]. The no qRT-PCR amplification of Glyma11g12980 is clarified in Fig B in S1 Supporting Information.

In the present study, we analyzed root RNA-seq data from flood-tolerant Embrapa 45 and flood-sensitive BR 4 soybean cultivars that showed contrasting grain yields when cultivated in waterlogged soil (Fig 1). In order to evaluate pairwise RNA-seq data, the relative expression of six common hypoxia-responsive genes [Trehalose-6-Phosphate Synthase (Glyma17g07530), Ascorbate Peroxidase (Glyma12g07780), Sucrose Synthase (Glyma13g17420), Alternative Oxidase (Glyma04g14800), non-symbiotic Hemoglobin (nsHB; Glyma11g12980), and Nitrate Reductase (Glyma06g11430)] were determined by qRT-PCR. The non-log-transformed qRT-PCR and RNA-seq expression data were consistent for all these genes (Fig 2) showing a strong positive Pearson correlation (r = 0.95; *P* < 0.001), indicating reliability in our transcriptome analysis.

### Transcriptome reconfiguration

#### Induction of signaling genes and down-regulation of genes related with energy-consuming processes under hypoxia

**Fig 3:**
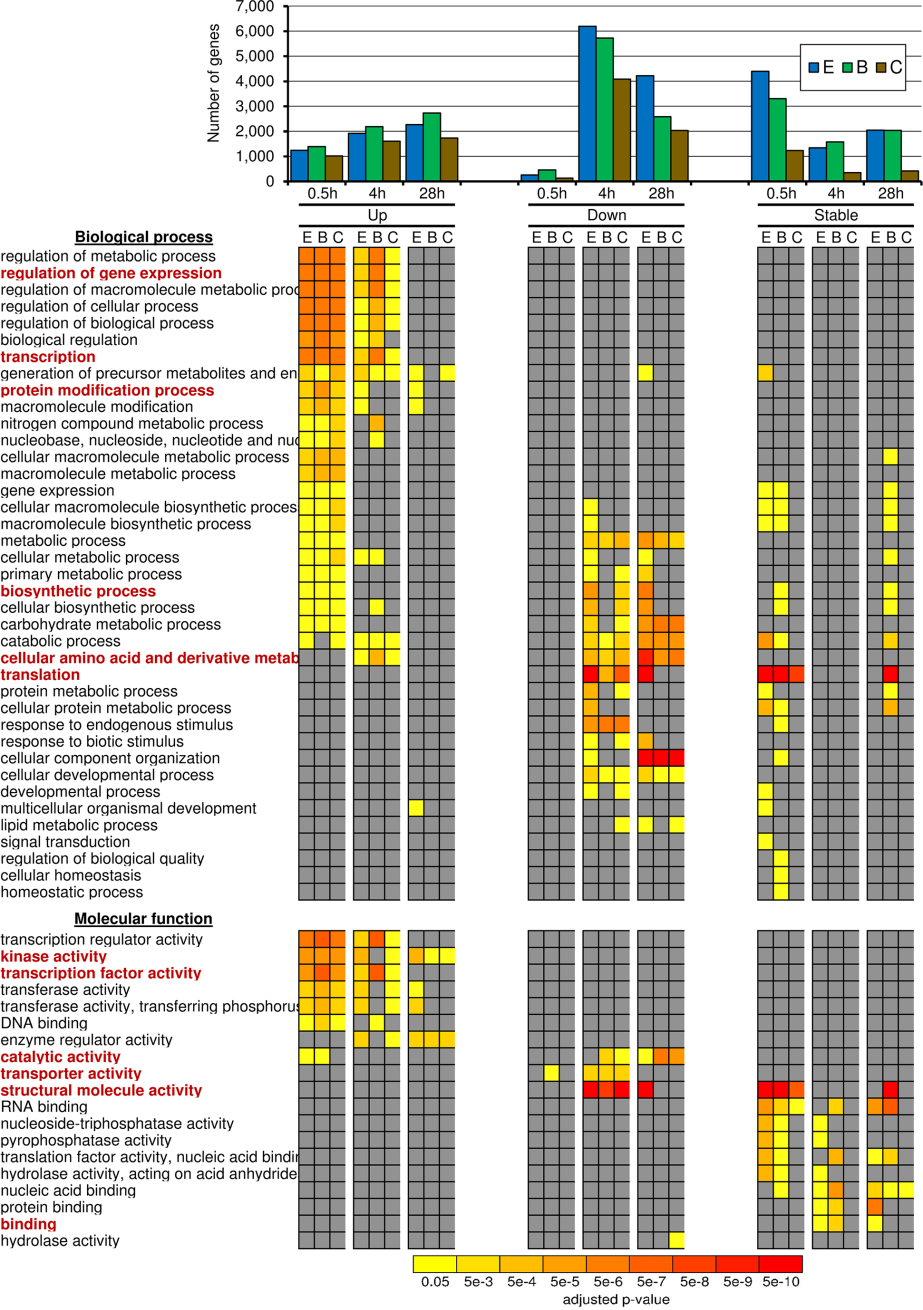
Number and GO enrichment analysis of up-, down-, and stable-regulated genes in flood-tolerant Embrapa 45 (E), flood-sensitive BR 4 (B), and in both soybean cultivars (C). Differentially expressed genes: fold-change ≥ 2 (up), ≤ -2 (down); adj. *p* ≤ 0.01; RPM ≥ 9 (control or stress datasets). Stable-regulated genes: fold-change ≤ 1.1 or ≥ -1.1; RPM ≥ 9 (control and stress datasets). RPM ≥ 9: at least 20 reads in all datasets. Red GO names are cited in the text.

From 54,174 predicted protein-coding genes in the soybean genome (assembly Glyma 1.1) [19], 2,656 up-regulated (URGs) and 4,970 down-regulated genes (DRGs) were found in both cultivars under hypoxic stress. Of these total, after 0.5, 4, and 28h of root hypoxia, 1,144, 5,687, and 3,761 genes were differentially expressed, of which 89, 28, and 46% were URGs, respectively (Fig 3). In *Arabidopsis thaliana*, another flood-sensitive species, more URGs were also observed in roots after 0.5h of hypoxic stress, and URGs were more prevalent deeper into stress conditions [27]. In contrast to our findings in soybean data, where DRGs number decreased after 28h, DRGs remained after 168h of waterlogging in roots of flood-tolerant gray poplar (*Populus × canescens*) [28]. Even so, Embrapa 45 showed fewer URGs and more DRGs than BR 4, of which the greatest difference in DEGs between cultivars was after 28h where Embrapa 45 (4,216 DRGs) had more DRGs than BR 4 (2,582 DRGs) (Fig 3).

Gene function was determined by identifying Gene Ontology (GO) categories for DEGs and SGs. The most enrichment for GO categories was found after 0.5h in URGs from both cultivars and after 4-28h in DRGs mainly from Embrapa 45 (Fig 3). Noteworthy GO categories in URGs were gene expression regulation (GO:0010468), more specifically for transcription (GO:0006350) and protein modification (GO:0006464) involving transcription factors (GO:0003700) and kinases (GO:0016301) activities. Overrepresented in DRGs were energy-demanding processes, including transport (GO:0005215) and biosynthesis (GO:0009058), as well as translation (GO:0006412), most of which encode ribosomal proteins (GO:0005198; structural molecule activity). Further, transcription factors (GO:0003700), kinases (GO:0016301) and transporters (GO:0005215) were enriched in DEGs, while more general functions such as binding (GO:0005488) in SGs. Our results show that hypoxia induces controlling/signaling genes and suppresses genes related with energy-consuming processes in soybean. Therefore, both induction and repression of genes may be important for flooding tolerance.

#### Hypoxic soybean roots experience changes in amino acids and amino acid-related metabolism

After 4h of hypoxia, URGs and DRGs were enriched for reorganization of cellular amino acid and derivative metabolic processes (GO:0006519) (Fig 3). Alterations in amino acid metabolism have been previously observed in hypoxic roots of soybean [29], *Lotus japonicus* [30], and gray poplar [28], including high accumulation of alanine and GABA (Gamma-Amino Butyric Acid) as well as reduction of aspartate level. In agreement, we observed up-regulation of alanine aminotransferase (Glyma07g05130) and glutamate decarboxylase (Glyma08g09670) at all-time points in both cultivars (S2 File).

Glutamate is directly related to alanine and aspartate metabolism via transamination, and to GABA via decarboxylation [31]. Interestingly, we observed induction of the genes NADH-dependent glutamate synthase (Glyma04g41540 and Glyma19g16486) and aspartate aminotransferase (Glyma14g13480, Glyma17g33050, Glyma06g08670, and Glyma01g32360), as well as repression of ferredoxin-dependent glutamate synthase (Glyma03g28410 and Glyma19g31120), ATP-dependent asparagine synthase (Glyma11g27480, Glyma11g27720, Glyma14g37440, and Glyma18g06840) (ArrayExpress database, accession number E-MTAB-5709). These responses suggests roots change NADH oxidation to save ATP for glutamate synthesis under hypoxia.

While expression of aspartate kinase, aspartate semialdehyde dehydrogenase and homocysteine S-methyltransferase increases under hypoxia, we observed repression of polyamines and phenylpropanoids biosynthesis-related genes, including upstream genes from shikimate pathway (Fig A in S1 Supporting Information, S2 File). Among DRGs was found S-adenosyl-L-methionine (SAM) synthase (EC 2.5.1.6). SAM connects to ethylene, polyamines, and phenylpropanoid-derived lignin pathways (Fig A in S1 Supporting Information) as well as histone and nucleic acid methylation for gene expression regulation [32,33]. Studies involving exogenous application or endogenous production of polyamines via genetic manipulation have shown increased tolerance to a broad spectrum of abiotic stresses [34], opening opportunities for improvement of soybean flooding tolerance by genetic engineering approaches.

#### Same gene, different regulation between cultivars: Identification of candidate genes for flooding-tolerance

**Fig 4:**
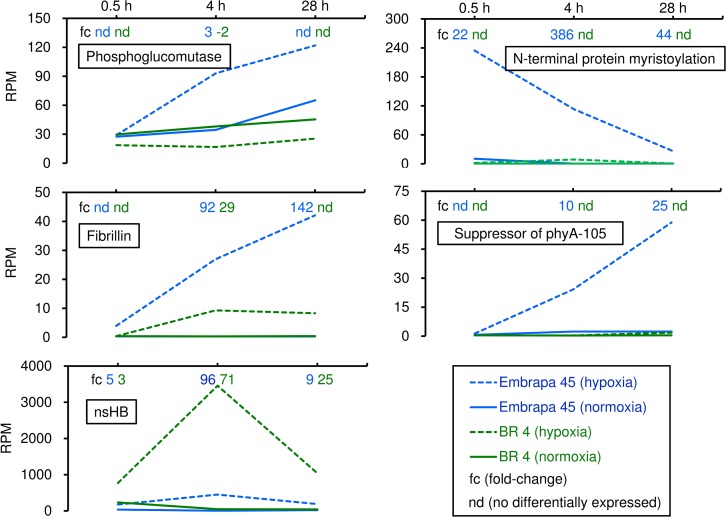
Relative expression (in fold-change) and expression level (in RPM) in flood-tolerant Embrapa 45 and flood-sensitive BR 4 soybean cultivars along three time points. The analyzed genes were phosphoglucomutase (Glyma05g34790), N-terminal protein myristoylation (Glyma06g03430), fibrillin (Glyma10g32620), suppressor of phyA-105 (Glyma06g37080), and non-symbiotic hemoglobin (nsHB; Glyma11g12980). Differentially expressed genes: fold-change ≥ 2 (up), ≤ -2 (down); adj. *p* ≤ 0.01; RPM ≥ 9 (control or stress datasets). RPM ≥ 9: at least 20 reads in all datasets.

The phosphoglucomutase (Glyma05g34790) gene was up-regulated in Embrapa 45 and down-regulated in BR 4 soybean cultivar after 4h of hypoxic stress (Fig 4). Its up-regulation in the flood-tolerant cultivar is in accordance with a shift in sucrose catabolism from ATP-dependent invertase-hexokinase to energy-saving SuSy-UGPase pathway [5]. In both cultivars, SuSy (Glyma19g40041, Glyma09g08550, and Glyma15g20180) and UGPase (Glyma11g33160) genes were observed up-regulated, while genes down-regulation were invertase (Glyma10g35890) and hexokinase (Glyma05g35890, Glyma07g12190, and Glyma17g37720) genes.

Higher induction of an unknown protein related to N-terminal protein myristoylation (Glyma06g03430), suppressor of phyA-105 (Glyma06g37080), and fibrillin (Glyma10g32620) were observed in flood-tolerant Embrapa 45. The *A*. *thaliana* ortholog of Glyma10g32620 (AT3G23400) is required for resistance to multiple stresses [35]. Moreover, differential expression of fibrillin genes correspond to plastoglobule number in leaves of contrasting soybean genotypes under drought and waterlogging stresses [36].

Although the nsHB gene (Glyma11g12980) exhibited similar expression ratio (in fold-change) between the two cultivars, the expression level (in RPM) in Embrapa 45 was lower under normoxic and hypoxic conditions (Fig 4). The last 284 nucleotides of the nsHB 3’UTR (3’Untranslated Region) are absent only in Embrapa 45 (Fig B in S1 Supporting Information). This explains the absence of qRT-PCR amplification in Embrapa 45 (Fig 2) and the similar transcriptional profile (qRT-PCR and RNA-seq) in both cultivars (Fig 2). Considering the important role of nsHB to protect plants under hypoxic stress [37], further study is required to understand the alternate 3’UTR structures influence transcription, transcript stability, and protein abundance.

### Function, structure, and composition of gene groups: Comparing its mechanistic relationship with expression regulation

To further understanding how soybean genes evolve and respond to hypoxia, the top 40 transcripts (all time points in both cultivars) and top 500 transcripts (at least one time point in both cultivars, except for S500 keeping all time points as criterion) stable and DEGs were structurally/compositionally characterized, comparing its mechanistic relationship with expression regulation.

#### The top gene groups differ functionally

As noted above, the genes aspartate aminotransferase (Glyma01g32360) and SAM decarboxylase (Glyma17g07830) were ranked in top 40 up-regulated (U40) and down-regulated (D40) groups, respectively. Likewise, phenylpropanoid/flavonoid related (D500) and SuSy-UGPase (U500) genes as well as three paralogs of Glyma06g03430 (N-terminal protein myristoylation) (U500) belonged to top 500 groups.

Genes associated with ethylene biosynthesis (ACC oxidase: Glyma05g36310), glycolysis (Pyruvate kinase: Glyma10g34490), ethanol fermentation (pyruvate decarboxylase: Glyma01g29190 and Glyma03g07380; alcohol dehydrogenase: Glyma04g39190 and Glyma14g27940), biotic stress defense (kunitz trypsin protease inhibitor: Glyma16g33770; polygalacturonase inhibiting protein: Glyma05g25370 and Glyma08g08360), and flooding governing acclimation (ERFVII related to *A*. *thaliana* HRE2: Glyma19g40070) were found in U40.

In the D40 were genes involved in antimicrobial defense (cysteine-rich secretory proteins: Glyma13g32500, Glyma13g32510, Glyma13g32530, and Glyma13g32540), gene regulation (bZIP: Glyma05g22860 and Glyma17g17100; MYB: Glyma13g20510; NAC: Glyma08g18470), oxygen consuming (2-oxoglutarate oxygenase: Glyma03g23770, Glyma07g12210, and Glyma08g18000; cytochrome P450: Glyma03g02410 and Glyma04g03790), and transport of dicarboxylate (Glyma07g39580), sulfite (Glyma07g30570), manganese (2+) and iron (2+) (Glyma08g08090, Glyma16g28340, and Glyma09g21920). These ions can accumulate to toxic levels in waterlogged plants [38–40].

The top 40 stable group (S40) has genes associated with protein (Glyma09g08830, Glyma06g01120, Glyma18g10060, Glyma18g32830, and Glyma04g23560) and nucleic acid binding (Glyma03g01920, Glyma18g11950, Glyma18g32190, Glyma06g48070, Glyma09g06750, Glyma12g29270, Glyma04g04880, and Glyma01g44460). For flooding stress, these genes are promising candidate reference genes for qRT-PCR normalization, given their higher stability compared to stable genes commonly used in the literature [17].

#### Top gene groups have different TATA box, ABRE, and DRE motif usage in promoter sequences. Does ABRE mediate gene expression independent of ABA in hypoxic soybean roots?

Transcription of protein-coding genes in eukaryotes requires numerous protein factors to recognize specific DNA loci. The core promoter region, proximal to the transcription start-site (TSS), recruits general transcription factors involved in basal transcription [41] and *cis*-regulatory elements from extended promoter recruits DNA-bound transcription factors (activators or repressors) to fine-tune the transcriptional control [42].

**Fig 5:**
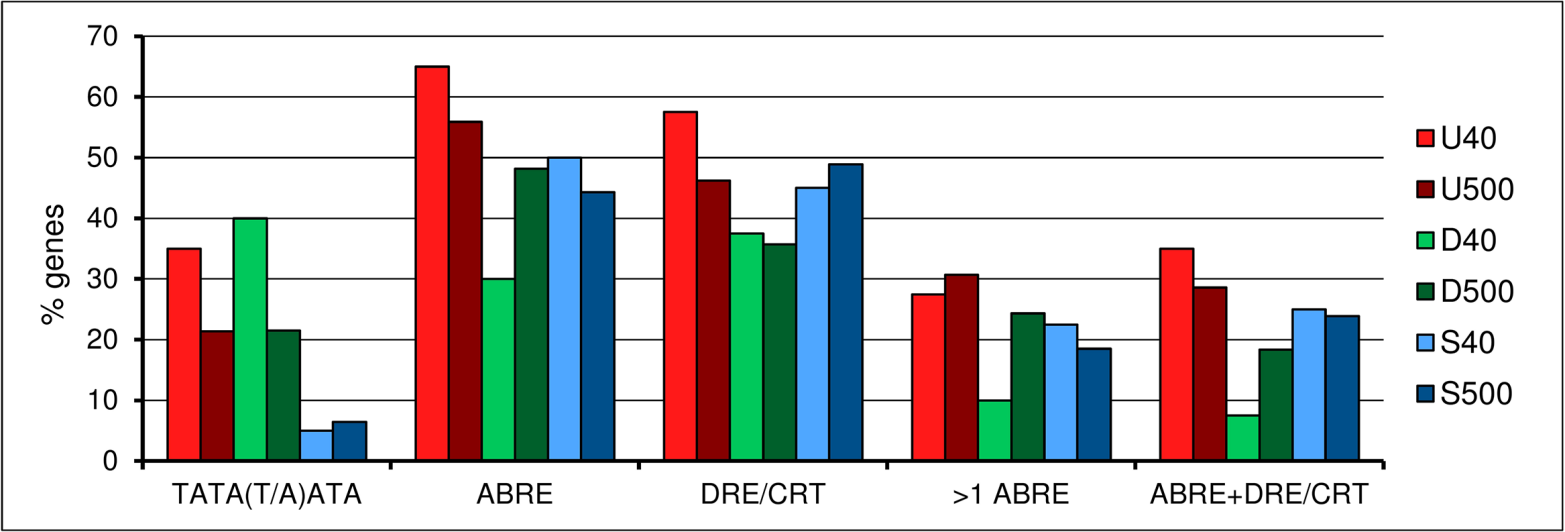
Percentage of genes with which putative promoter region contain consensus TATA box, ABRE, and/or DRE/CRT motifs. Statistical significances from pairwise comparisons are provided in Table B in S1 Supporting Information.

The general regulator TATA-box binding protein (TBP) is required for transcription initiation by all three eukaryotic RNA polymerases [43]. TBP can bind to various DNA sequences but has higher affinity for the consensus TATA-box [44]. Based on previous work [45–47], we scanned for the core sequence TATA extending 4 bp in the 3' direction within the 50 bp region upstream of the predicted TSS (between -50 and -1). We found more DEGs (from 21% in U500 to 40% in D40) with the consensus TATA box sequence TATA(T/A)ATA than SGs (at most 6% in S500) (Fig 5, Table B in S1 Supporting Information). Our results are in agreement with hexamer sequences TATA(T/A)A over-represented in *A*. *thaliana*, *Oryza sativa*, and *Glycine max* genomes [48]. Similarly, Tirosh et al. [45] observed a correlation between consensus TATA-containing genes being differentially expressed and TATA less-containing genes stable expressed in yeast, metazoans, and plants.

Higher TBP turnover at consensus TATA- compared to TATA-less promoters is associated with specific coactivators [46,49]. Coactivators are multisubunit complexes represented by SAGA (Spt-Ada-Gcn5-Acetyltransferase), TFIID (transcription Factor II D), related with TBP binding on TATA and TATA-less promoters, respectively [49], and Mediator [50]. The latter is organized into head, middle, tail, and Cdk8 kinase modules to converge and transmit signals from sequence-specific transcription factors to RNA polymerase [51,52]. Here, mediator components Glyma13g16910 (head MED20a) and Glyma13g31480 (tail MED16) were at least three times down- and nine times up-regulated after 4 and 28h of hypoxia, respectively, in both cultivars. The head module MED20a subunit participates in transcription regulation of miRNA and protein-coding genes involved with plant development, time flowering, and fruit size in *A*. *thaliana* [53]. In contrast, the tail module MED16 component regulates several biotic [54,55] and abiotic [56–58] stress responses in plants. Med16 is required for transcriptional activation of cold- and dehydration-inducible genes that have C-repeat/dehydration-responsive elements (CRT/DRE) promoter motifs controlled by CRT/DRE-binding transcription factors (CBF/DREB) [57,58].

The CRT/DRE and CBF/DREB, the *cis*-acting ABA-responsive elements (ABRE) and corresponding *trans*-acting factors ABRE-binding proteins/ABRE-binding factors (AREB/ABF) play important roles in abiotic stress tolerance in plants [59]. Based on the genomatix database (http://www.genomatix.de/) [60], we found that *cis*-acting ABRE and DRE/CRT motifs were most frequent in the putative promoter region (1000 bp up- and 100 bp down-stream the TSS) of top up- compared to top down-regulated genes (Fig 5, Table B in S1 Supporting Information). The AREB/ABF genes Glyma06g04353 and Glyma19g30230 increased their mRNA abundance (~2 fc) after 28h of hypoxia. The higher differential expression of GmDREB1B;1 (Glyma20g29410 [61]) and of GmDREB2A;2 (Glyma14g06080 [62]) was observed after 0.5h (>20 fc) and 28h (>24 fc) of hypoxia, respectively. Both these genes are also induced by heat, cold, drought, and salinity stress, and up-regulate ABA receptor GmPYL21 (Glyma13g30210 [63]) [61,62]. GmPYL14 (Glyma14g06100, up-regulated by GmDREB2A;2 [62]), and GmPYL21 were up-regulated under hypoxia (>9 and >3 fc after 4h, respectively). These receptors interact with the phosphatase GmPP2C1 (Glyma13g16640, at most 3 fc under hypoxia), inhibiting it in an ABA independent manner [63]. Interestingly, Kidokoro et al. [61] observed that transcriptional activation of GmPYL21 by GmDREB1B;1 can enhance ABRE-mediated gene expression in an ABA-independent manner under cold stress, although ABA levels are not increased under such condition. We propose that ABRE-mediated gene expression is ABA-independent in hypoxic soybean roots. Although exogenous application of ABA improves flooding tolerance of plants [64–66], endogenous ABA decreases in roots under flooding stress [67–69]. We observed down-regulation of genes related to ABA biosynthesis (Glyma19g06540, Glyma06g08944, Glyma13g27220, Glyma11g21160, and Glyma14g04950) and up-regulation of ABA inactivation genes GmCYP707As (Glyma16g20490, Glyma01g35660, and Glyma09g35250 [70]). In addition to GmCYP707As, hypoxia changes expression of soybean orthologs of *A*. *thaliana* genes involved in ABA signaling (ABA receptors Pyl4-6; phosphatases ABI1-2, HAB1-2, HAI1-3, and AHG3; AFP1-4; MAPKKK18) dependent on SRK2D/E/I (SNF1-related kinases SRK2D/SnRK2.2, SRK2E/SnRK2.6, and SRK2I/SnRK2.3) (Genevestigator microarray database [71]) (S3 File). SRK2D/E/I are key activators of AREB/ABF proteins [72]. Glyma01g39020, which is most similar in amino acid sequence to SRK2D/E/I, is up-regulated 2- and 3-fold after 4 and 28h of hypoxia. Moreover, orthologous genes of CIPK-6 and CIPK-25 (Glyma17g08270, Glyma04g06520, and Glyma06g06550) were up-regulated at all-time points (from 4 to 40 fc) under hypoxia. These are kinases involved in Ca^+2^-mediated expression of DREB1-2 and ABA signaling [73,74]. Strikingly, although ABI1-2, PP2CA, and HAI1-2 genes from *A*. *thaliana* are down-regulated under hypoxic stress, we observed strong up-regulation of soybean orthologs of Glyma01g43460 (HAI3) transcripts at all-time points (S3 File). These *A*. *thaliana* genes are up-regulated under drought, osmotic, and salinity stresses (S3 File) and soybean genes under drought stress [18], stresses associated with ABA production. In agreement with a role for AREB in tolerance of diverse abiotic stresses, our results indicate although ABA level decreases in soybean roots under hypoxia, ABRE-mediated gene expression may occur. In this context, AREB/ABF are powerful candidates to improve flooding tolerance.

#### Top gene groups differed in structure, composition, and codon usage. How may hypoxia alter translation?

**Fig 6:**
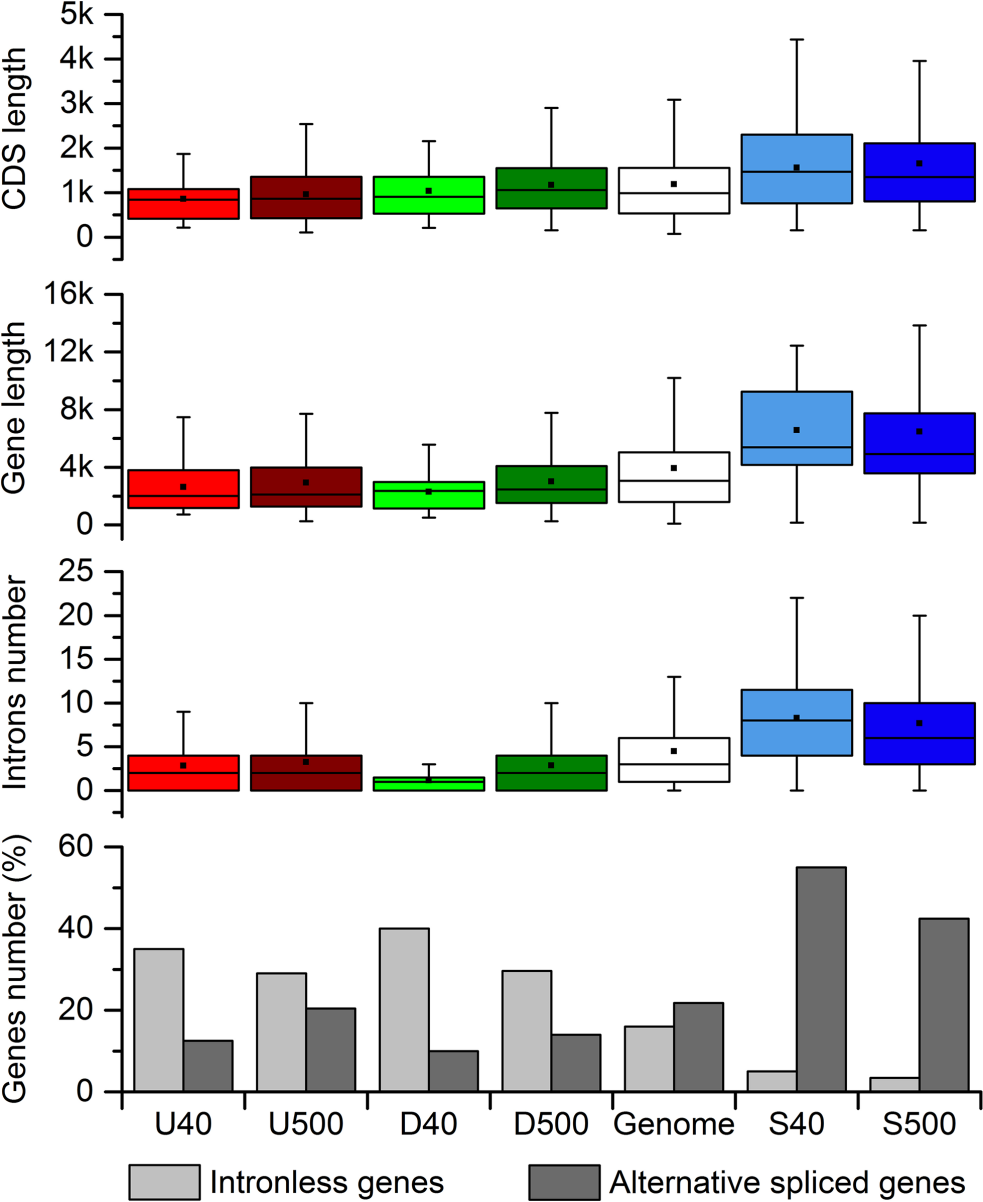
Structural features among gene groups. The groups are formed by top 40 and 500 ranked up- (U), down-regulated (D), and stable-genes (S) to hypoxia, and genome. The box is determined by the 25th and 75th percentiles with a line as the median and a black square as the mean of the data. Error bars extend 1.5 times the interquartile range from the 25th and 75th percentiles. Statistical significances from pairwise comparisons are provided in Tables B and C in S1 Supporting Information.

Compared to SGs, DEGs are smaller, with shorter CDS (coding sequence) length and fewer introns (Fig 6, Table C in S1 Supporting Information). Similar results were observed in *A*. *thaliana* [75], yeasts, and mice [76] for genes responsive to other stresses. Shorter genes with fewer introns demand less energy [77] and can have faster expression dynamics [78]. Interestingly, SGs also seem to have improvement of energetic and time costs. We compared soybean SGs with cognate proteins [79], and analyzed *A*. *thaliana* data sets of immunopurified polysomal mRNAs [80] and translationally inactive mRNAs [81]. The results suggest that energy is saved from translation by down-regulation of cognate soybean proteins under hypoxia (Fig C in S1 Supporting Information). In this context, stable mRNAs from *A*. *thaliana* are sequestered into stress granules and poorly associated with translating ribosomes under hypoxia. Upon reoxygenation, they are rapidly released from stress granules forming new polyribosomes, minimizing the need for *de novo* transcription.

The higher intron number and number of splicing variants in soybean SGs (Fig 6, Tables B and C in S1 Supporting Information) are in agreement with the higher gene body (i.e., transcript region) methylation in SGs [75,82,83], involved in splicing regulation [84–86]. Here, whereas SGs exhibited steeper 5’ to 3’ negative G+C and CpG gradient, no decrement from start to middle region of DEGs were observed (Fig D and Table C in S1 Supporting Information). The low strength but diverged G+C and CpG patterns among gene groups can be influenced by gene structure, recombination, and DNA methylation [87]. Moreover, C3pG1 (C at third position of a codon binding G at first position of a neighbor codon) and G1 content were higher among di- and mono-nucleotides, respectively, and C3 was the mono-nucleotide with more diverged content among gene groups, mainly at CDS middle region.

**Fig 7:**
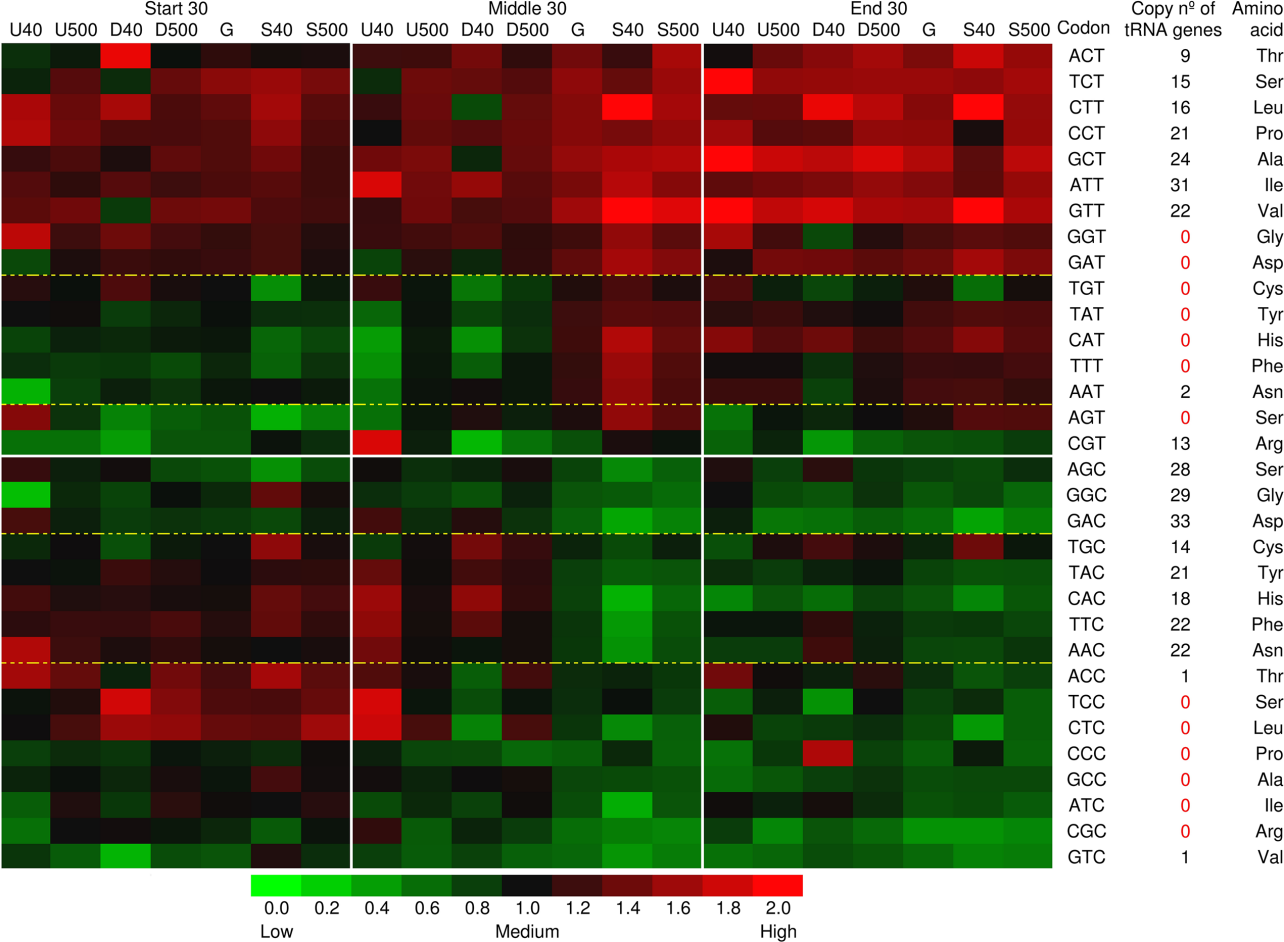
Heat map of relative synonymous codon usage (RSCU) for start, middle, and end 30 CDS (coding sequence) codons ending in pyrimidine (C and T) from different soybean gene groups. The groups are formed by top 40 and 500 ranked up- (U), down-regulated (D), and stable-genes (S) to hypoxia, and genome (G). Asp, Cys, Tyr, His, Phe, and Asn are coding by 2-fold degenerate pyrimidine ending codons. Heat map of RSCU for all 59 synonymous codons including for whole CDS extension is provided in Fig E in S1 Supporting Information.

The relative synonymous codon usage (RSCU) analysis for all 59 synonymous codons showed that C3 content divergences highlighted in 2-fold degenerate pyrimidine ending codons between CDS edges as well as among gene groups at CDS middle region (Fig 7). It is noteworthy that 2-fold degenerate codons ending in pyrimidines seem to be in majority [maybe exclusively, including in soybean (Fig E in S1 Supporting Information)] decoded by G34 tRNAs (G at position 34 of the tRNA, the first anticodon position) in all three domains of life (archaea, bacteria, and eukarya) [88,89]. This suggests strong positive selection to discriminate correct cognate C3 and wobble U3 codons from the incorrect near-cognate codons G3 and A3 (e.g., CAC/U histidine versus CAG/A glutamine). C3 over U3 2-fold degenerate ending codon bias also occurs at evolutionarily conserved amino acids sites across 12 fly drosophilid species and correspond to higher levels of G34-to-Q34 substitution [90]. This opens a question if Q34 tRNA influences the compositional divergence among soybean gene groups. Remarkably, queuine (q), the free base of Q (queuosine), is only synthesized by bacteria and salvaged by most eukaryotes [91]. Example is the legume model *Medicago truncatula*, in which rhizobium Q synthesis is required for effective nitrogen-fixing symbiosis [92]. In contrast, this is not observed in Brassicaceae, including *A*. *thaliana*, given the absence of genes encoding transglycosylases that catalyze q insertion in target G34U35N36 tRNAs (N = any base), found in *Medicago* [91] and soybean (Glyma04g10706, Glyma01g40041, Glyma13g10281, Glyma11g05250, Glyma06g10555, and Glyma08g48310). Besides Q, wybutosine (yW) is another hypermodified nucleoside derived from G, but yW is found exclusively at position 37 (neighboring anticodon sequence) of tRNAs that decode phenylalanine (codons UUU and UUC; biased here among soybean gene groups), important to reduce polyuridine translational frameshift errors [93].

Here, DEGs related to modification of inosine, queuosine, wybutosine, pseudouridine, and methylation (tRNA and DNA methyltransferases), as well as aminoacylation of tRNAs (amino acid charging) were observed under hypoxia (S4 File). In addition, our results indicate that hypoxia impairs biosynthesis of methionine, mainly SAM, and consequently DNA and tRNA methylation (Fig A in S1 Supporting Information, S2 File). Although S-methylmethionine cycle seems to alleviate methionine decrement by transcriptional up-regulation of homocysteine S-methyltransferase (ec: 2.1.1.10), genes encoding folate-dependent methionine synthase (ec: 2.1.1.14) as well as SAM synthase (EC 2.5.1.6) and SAH hydrolase (EC 3.3.1.1) were down-regulated under hypoxic stress (Fig A in S1 Supporting Information, S2 File). This can also occur at protein level [79].

Differential expression of aminoacyl-tRNA synthetases (aaRS) also occur in soybean leaf under drought stress, with more aaRS DEGs in wild-type than in transgenic lines overexpressing BiP chaperone [94]. The BiP (Binding Protein) major regulates the endoplasmic reticulum (ER) stress [95]. Here, many ER stress related genes [96] were differentially expressed (S5 File), including down-regulation of BiP (Glyma05g36600, Glyma05g36620, Glyma08g02940, and Glyma08g02960; from -2 to -10 fc after 4 and 28h in both cultivars under hypoxia). Among these aaRS differentially expressed, Glyma14g11711 (aspartyl-tRNA synthetase) was up-regulated at all-time points in the two cultivars (from 5 to 16 fc). Curiously, transgenic plants overexpressing an aspartyl tRNA synthase (AspRS) orthologue (At4g31180) improve tolerance to biotic stress [97]. The At4g31180 is target of a synthetic isomer of GABA, called BABA (β-Amino Butyric Acid) [97]. BABA primes plants to enhance tolerance against broad-spectrum of biotic and abiotic stresses [98]. Inhibition of AspRS activity by BABA accumulates uncharged tRNA Asp, which as others uncharged tRNAs is signaling molecule to attenuate translation by Gcn2-eIF2α system, and subsequently alleviate ER stress [99]. Based on this, further studies may help to elucidate involvement of amino acids metabolism in tRNA modifications, translation accuracy/efficiency, and ER stress under hypoxia. In addition, BiP and aaRS are candidates for biotechnology applications for improvement in flooding tolerance.

## Supporting information

S1 Supporting Information **File containing all supporting Tables (A-D) and Figures (A-E). Table A in S1 Supporting Information.** Samples sizes used for structural and compositional analysis among groups of genes. **Table B in S1 Supporting Information.** Fisher’s pairwise comparisons between gene groups. **Table C in S1 Supporting Information.** Mann-Whitney pairwise comparisons. **Table D in S1 Supporting Information.** Primer pairs used in the study. **Fig A in S1 Supporting Information.** KEGG pathways showing transcriptional changes for amino acids and derivative metabolic process in both Embrapa 45 and BR 4 soybean cultivars. **Fig B in S1 Supporting Information.** Structural divergence of non-symbiotic hemoglobin Glyma11g12980.1 transcript between BR 4 and Embrapa 45 cultivars by mapping (A) and *de novo* assembly (B) of reads. **Fig C in S1 Supporting Information.** Steady-state stable mRNAs tend to be translationally down-regulated (A), decreasing their association with ribosomes (of which include RPL18 component) by their sequestration into stress granules (ribonucleoprotein complexes including UBP1C) under hypoxia, whereas upon reoxygenation they are rapidly released from stress granules forming new polyribosomes (B). **Fig D in S1 Supporting Information.** Compositional features for full (normalized to 90 nucleotides), start, middle, and end 90 nucleotides length from coding sequences from different soybean gene groups. **Fig E in S1 Supporting Information.** Heat map of RSCU (Relative Synonymous Codon Usage) for full, first, middle, and last 30 CDS (coding sequence) codons length from different soybean gene groups.
(PDF)

S1 File Tab-delimited file showing raw data for gene expression from flood-tolerant Embrapa 45 (E) and flood-sensitive BR 4 (B) soybean cultivars under normoxia (N) and hypoxia (H) along 0.5, 4, and 28h. Top 40 and 500 ranked up- (U), down-regulated (D), and stable-genes (S) are shown in separate excel sheets named accordingly.
(XLSX)

S2 File Differentially expressed genes (DEGs) related with amino acids and derivative metabolic process in hypoxic roots of flood-tolerant Embrapa 45 (E) and flood-sensitive BR 4 (B) soybean cultivars along 0.5, 4, and 28h. DEGs: fold-change ≥ 2 (up), ≤ -2 (down); adj. p ≤ 0.01; RPM ≥ 9 (control or stress datasets). RPM ≥ 9: at least 20 reads in all datasets.
(XLSX)

S3 File Differentially expressed genes (DEGs) from hypoxic roots of flood-tolerant Embrapa 45 (E) and flood-sensitive BR 4 (B) soybean cultivars along 0.5, 4, and 28h, and *Arabidopsis thaliana* orthologs related with ABA metabolism and signaling dependent of SRK2D/E/I responding to diverse treatments (from Genevestigator microarray database) [71]. (XLSX)

S4 File Differentially expressed genes (DEGs) related with modification of I34, Q34, yW37, pseudouridine, and methylation (tRNA and DNA methyltransferases), as well as aminoacylation of tRNAs (amino acid charging) in hypoxic roots of flood-tolerant Embrapa 45 (E) and flood-sensitive BR 4 (B) soybean cultivars along 0.5, 4, and 28h. (XLSX)

S5 File Differentially expressed genes (DEGs) related with endoplasmic reticulum stress [unfolded protein response (UPR) and ER stress-induced plant-specific cell death signaling pathways] [96] analyzed in hypoxic soybean roots of flood-tolerant Embrapa 45 (E) and flood-sensitive BR 4 (B) soybean cultivars along 0.5, 4, and 28h. DEGs: fold-change ≥ 2 (up), ≤ -2 (down); adj. p ≤ 0.01; RPM ≥ 9 (control or stress datasets). RPM ≥ 9: at least 20 reads in all datasets.
(XLSX)

## Abbreviations

aaRS: aminoacyl-tRNA synthetases
ABRE: *cis*-acting ABA-responsive element
BABA: β-amino butyric acid
BiP: chaperone binding protein
CRT/DREs: *cis*-acting C-repeat/dehydration-responsive elements
DEGs: differentially expressed genes
DRGs: down-regulated genes
ER: endoplasmic reticulum
fc: fold-change
GABA: gamma-aminobutyric acid
GO: gene ontology
QTLs: quantitative trait loci
qRT-PCR: quantitative real-time PCR
RPM: reads per mapped million
RSCU: relative synonymous codon usage
SAGA: spt-ada-gcn5-acetyltransferase
SAH: S-adenosyl-L-homocysteine
SAM: S-adenosyl-L-methionine
SGs: stable genes
TBP: TATA-box binding protein
TFIID: transcription Factor II D
TSS: transcription start-site
URGs: up-regulated genes
