# Supplementary material for: Insights into soybean transcriptome reconfiguration under hypoxic stress: Functional, regulatory, structural, and compositional characterization
Source: PLoS One. 2017 Nov 16;12(11):e0187920. doi: 10.1371/journal.pone.0187920 (PMC5690659; doi:10.1371/journal.pone.0187920)
Supplement: S1 Supporting Information — File containing all supporting Tables (A-D) and Figures (A-E). Table A in S1 Supporting Information. Samples sizes used for structural and compositional analysis among groups of genes. Table B in S1 Supporting Information. Fisher’s pairwise comparisons between gene groups. Table C in S1 Supporting Information. Mann-Whitney pairwise comparisons. Table D in S1 Supporting Information. Primer pairs used in the study. Fig A in S1 Supporting Information. KEGG pathways showing transcriptional changes for amino acids and derivative metabolic process in both Embrapa 45 and BR 4 soybean cultivars. Fig B in S1 Supporting Information. Structural divergence of non-symbiotic hemoglobin Glyma11g12980.1 transcript between BR 4 and Embrapa 45 cultivars by mapping (A) and de novo assembly (B) of reads. Fig C in S1 Supporting Information. Steady-state stable mRNAs tend to be translationally down-regulated (A), decreasing their association with ribosomes (of which include RPL18 component) by their sequestration into stress granules (ribonucleoprotein complexes including UBP1C) under hypoxia, whereas upon reoxygenation they are rapidly released from stress granules forming new polyribosomes (B). Fig D in S1 Supporting Information. Compositional features for full (normalized to 90 nucleotides), start, middle, and end 90 nucleotides length from coding sequences from different soybean gene groups. Fig E in S1 Supporting Information. Heat map of RSCU (Relative Synonymous Codon Usage) for full, first, middle, and last 30 CDS (coding sequence) codons length from different soybean gene groups. (PDF) [file pone.0187920.s001.pdf]

S1 Supporting Information

Insights into soybean transcriptome reconfiguration under hypoxic stress: functional, regulatory, structural, and compositional characterization

Thiago J. Nakayama[1], Fabiana A. Rodrigues[2], Norman Neumaier[2], Juliana Marcolino-Gomes[2], Hugo B. C. Molinari[3], Thaís R. Santiago[3], Eduardo F. Formighieri[3], Marcos F. Basso[3], José R. B. Farias[2], Beatriz M. Emygdio[4], Ana C. B. de Oliveira[4], Ângela D. Campos[4], Aluízio Borém[1], Frank G. Harmon[5], Liliane M. Mertz-Henning[2], Alexandre L. Nepomuceno[2*]

[1]Departamento de Fitotecnia, Universidade Federal de Viçosa, Viçosa, Minas Gerais, Brazil
[2]Embrapa Soja, Empresa Brasileira de Pesquisa Agropecuária, Londrina, Paraná, Brazil
[3]Embrapa Agroenergia, Empresa Brasileira de Pesquisa Agropecuária, Brasília, Distrito Federal, Brazil
[4]Embrapa Clima Temperado, Empresa Brasileira de Pesquisa Agropecuária, Pelotas, Rio Grande do Sul, Brazil
[5]Department of Plant and Microbial Biology, University of California-Berkeley, Berkeley, California, USA

*Corresponding author
alexandre.nepomuceno@embrapa.br (ALN)

S1 Supporting Information

Table A. Samples sizes used for structural and compositional analysis among groups of genes.

Table B. Fisher's pairwise comparisons between gene groups.

Table C. Mann-Whitney pairwise comparisons.

Table D. Primer pairs used in the study.

Fig A. KEGG pathways showing transcriptional changes for amino acids and derivative metabolic process in both Embrapa 45 and BR 4 soybean cultivars.

Fig B. Structural divergence of non-symbiotic hemoglobin Glyma11g12980.1 transcript between BR 4 and Embrapa 45 cultivars by mapping (A) and *de novo* assembly (B) of reads.

Fig C. Steady-state stable mRNAs tend to be translationally down-regulated (A), decreasing their association with ribosomes (of which include RPL18 component) by their sequestration into stress granules (ribonucleoprotein complexes including UBP1C) under hypoxia, whereas upon reoxygenation they are rapidly released from stress granules forming new polyribosomes (B).

Fig D. Compositional features for full (normalized to 90 nucleotides), start, middle, and end 90 nucleotides length from coding sequences from different soybean gene groups.

Fig E. Heat map of RSCU (Relative Synonymous Codon Usage) for full, first, middle, and last 30 CDS (coding sequence) codons length from different soybean gene groups.

Table A. Samples sizes used for structural and compositional analysis among groups of genes.

gene groups	promotor cis elements	gene structure	CDS			
			CpG		C1pG2, C2pG3, C3pG1	
			full	start 90, middle 90, end 90	full	start 90, middle 90, end 90
U40	40	40	40	34	40	34
U500	381	500	500	424	500	424
D40	40	40	40	37	40	37
D500	386	500	500	467	500	467
Genome	---	54174	54174	46644	10835	9329
S40	40	40	40	38	40	38
S500	352	500	500	490	500	490

genes groups	promotor cis elements	gene structure	CDS			
			G+C		C1, C2, C3, G1, G2, G3	
			full	start 90, middle 90, end 90	full	start 90, middle 90, end 90
U40	40	40	40	34	40	34
U500	381	500	500	424	500	424
D40	40	40	40	37	40	37
D500	386	500	500	467	500	467
Genome	---	54174	54174	46644	10835	9329
S40	40	40	40	38	40	38
S500	352	500	500	490	500	490

Table B. Fisher’s pairwise comparisons between gene groups.

	Promoters (%) with					Genes (%)	
	Consensus TATA-Box TATA(T/A)ATA	P$ABRE	P$DREB	ABRE >1	P$ABRE+P$DREB	Intronless	Alternative splicing
U40 vs D40	0.82	**0.00**	0.12	0.08	**0.01**	0.82	1.00
U40 vs S40	**0.00**	0.26	0.37	0.80	0.46	**0.00**	**0.00**
D40 vs S40	**0.00**	0.11	0.65	0.22	0.07	**0.00**	**0.00**
U500 vs D500	1.00	0.04	**0.00**	0.05	**0.00**	0.89	**0.01**
U500 vs S500	**0.00**	**0.00**	0.51	**0.00**	0.15	**0.00**	**0.00**
D500 vs S500	**0.00**	0.30	**0.00**	0.06	0.07	**0.00**	**0.00**

Table C. Mann-Whitney pairwise comparisons.

	**Introns number**	**Gene length (pb)**	**CDS length (pb)**
U40 vs D40	0.04	0.74	0.26
S40 vs U40	**0.00**	**0.00**	**0.00**
S40 vs D40	**0.00**	**0.00**	**0.01**
U500 vs D500	0.46	0.02	**0.00**
S500 vs U500	**0.00**	**0.00**	**0.00**
S500 vs D500	**0.00**	**0.00**	**0.00**

	**CDS G+C norm 90**	**Start 90 G+C**	**Middle 90 G+C**	**End 90 G+C**
U40 vs D40	0.71	0.15	0.34	0.27
S40 vs U40	0.01	0.27	**0.00**	0.69
S40 vs D40	0.08	0.04	0.05	0.51
U500 vs D500	**0.00**	0.54	0.02	0.02
S500 vs U500	0.09	**0.00**	**0.00**	**0.00**
S500 vs D500	**0.00**	**0.00**	**0.00**	0.55

	**CDS CpG norm 90**	**Start 90 CpG**	**Middle 90 CpG**	**End 90 CpG**
U40 vs D40	0.53	0.65	0.82	0.10
S40 vs U40	0.98	**0.00**	0.16	0.45
S40 vs D40	0.53	**0.00**	0.62	0.29
U500 vs D500	0.58	**0.00**	0.51	0.13
S500 vs U500	0.09	**0.00**	**0.00**	0.93
S500 vs D500	0.35	**0.00**	**0.00**	0.10

Table C. (continued).

	CDS norm 90			Start 90			Middle 90			End 90		
	**C1pG2**	**C2pG3**	**C3pG1**	**C1pG2**	**C2pG3**	**C3pG1**	**C1pG2**	**C2pG3**	**C3pG1**	**C1pG2**	**C2pG3**	**C3pG1**
U40 vs D40	0.89	0.59	0.54	0.76	0.31	0.50	0.66	0.14	0.43	0.38	0.26	0.75
S40 vs U40	**0.00**	0.23	0.18	**0.00**	0.10	0.11	0.52	0.96	0.03	0.19	0.03	0.53
S40 vs D40	**0.01**	0.93	0.50	**0.00**	0.02	0.21	0.88	0.11	0.17	0.03	0.20	0.82
U500 vs D500	0.02	0.99	0.06	0.06	0.04	0.20	**0.00**	0.23	0.99	0.10	0.11	0.50
S500 vs U500	**0.00**	0.06	0.02	**0.00**	**0.00**	**0.00**	0.86	**0.00**	**0.00**	**0.00**	0.01	0.04
S500 vs D500	**0.00**	0.08	**0.00**	**0.00**	**0.00**	**0.00**	**0.00**	**0.00**	**0.00**	**0.00**	0.34	**0.01**

	**Start 90 G+C vs Middle 90 G+C**	**Start 90 G+C vs End 90 G+C**	**Middle 90 G+C vs End 90 G+C**
S40	**0.00**	**0.01**	0.22
D40	0.77	0.22	0.11
U40	0.34	0.02	**0.01**
S500	**0.00**	**0.00**	**0.01**
D500	0.34	**0.00**	**0.00**
U500	0.04	**0.00**	**0.00**

	**Start 90 CpG vs Middle 90 CpG**	**Start 90 CpG vs End 90 CpG**	**Middle 90 CpG vs End 90 CpG**
S40	**0.00**	**0.00**	0.60
D40	0.61	0.19	0.12
U40	0.61	0.65	0.22
S500	**0.00**	**0.00**	0.03
D500	0.09	0.05	**0.00**
U500	0.59	**0.00**	**0.00**

Table C. (continued).

	CDS norm 90						First 90					
	**C1**	**G2**	**C2**	**G3**	**C3**	**G1**	**C1**	**G2**	**C2**	**G3**	**C3**	**G1**
U40 vs D40	0.72	0.62	0.84	0.75	0.64	0.54	0.53	0.52	0.98	0.57	0.97	0.06
S40 vs U40	0.35	0.77	0.70	0.10	**0.00**	0.73	0.49	0.53	0.77	0.05	0.73	0.13
S40 vs D40	0.62	0.95	0.70	0.04	**0.00**	0.31	0.98	0.27	0.92	0.07	0.87	0.46
U500 vs D500	**0.00**	**0.00**	**0.00**	**0.01**	**0.00**	0.77	0.14	0.85	0.02	0.08	**0.00**	0.10
S500 vs U500	**0.00**	0.22	0.75	0.23	**0.00**	**0.00**	0.45	0.82	0.03	0.90	0.28	0.15
S500 vs D500	0.13	0.05	**0.00**	0.07	**0.00**	**0.00**	0.48	0.66	0.82	0.08	0.05	0.83

	Middle 90						End 90					
	**C1**	**G2**	**C2**	**G3**	**C3**	**G1**	**C1**	**G2**	**C2**	**G3**	**C3**	**G1**
U40 vs D40	**0.01**	0.16	0.04	**0.00**	**0.01**	0.68	0.38	0.39	0.84	0.14	0.07	0.06
S40 vs U40	0.08	0.63	0.05	0.14	**0.00**	0.20	0.18	0.01	0.49	0.37	0.01	0.04
S40 vs D40	0.35	0.26	0.95	0.02	**0.00**	0.35	0.61	0.23	0.33	0.58	**0.00**	**0.00**
U500 vs D500	0.84	0.29	0.05	0.50	0.10	0.87	0.62	0.68	0.77	0.76	0.02	0.89
S500 vs U500	0.13	0.77	0.42	0.56	**0.00**	**0.01**	0.42	**0.00**	0.06	1.00	**0.00**	**0.00**
S500 vs D500	0.18	0.44	0.25	0.22	**0.00**	0.01	0.73	**0.00**	0.03	0.74	**0.00**	**0.00**

Table D. Primer pairs used in the study.

Name	Gene locus	Forward primer sequence	Reverse primer sequence
SUSY	Glyma13g17420	TGTTGTTGCATGATTTGGATCTTG	CACGGCTTAAAATTGAATTGATGG
NR	Glyma06g11430	AATCCCATGCAAGCTCATCTCC	CAAACCCATGAGTAGGTCGTCG
nsHB	Glyma11g12980	TGAAACCACCCTCCTCTTAGACTCC	GAATGCGAAACACTTCCCAACC
AOX	Glyma04g14800	CTTCTTGGGGTATTTGTTGTCACCT	ATTGCCCTTGTCCAGCTCCTTA
APX	Glyma12g07780	CACGGTGCCCATAATATTTCTCTC	CAACCCAACTCCAATCATCATCAC
TPS	Glyma17g07530	TGCGTAACAGCAATCTTTCAACAA	CCCAAGACCTAATCCACCAACC
ELF1B[A]	Glyma02g44460	GTTGAAAAGCCAGGGGACA	TCTTACCCCTTGAGCGTGG
ACTB[B]	Glyma15g05570	GAGCTATGAATTGCCTGATGG	CGTTTCATGAATTCCAGTAGC

Primers obtained from: A [100], B [101].

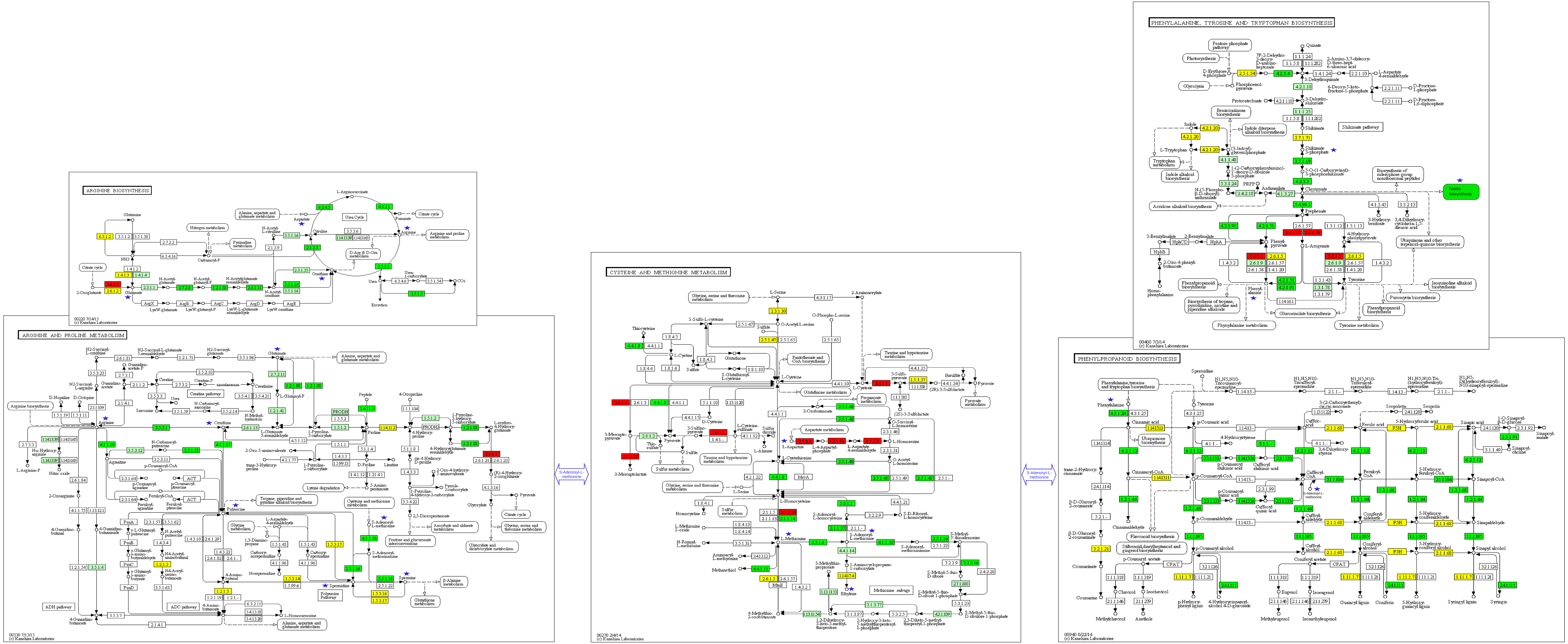

Fig A. KEGG pathways showing transcriptional changes for amino acids and derivative metabolic process in both Embrapa 45 and BR 4 soybean cultivars. Oriented by blue star icons, although up-regulation (red boxes) of some Asp catabolic related genes, many down-stream related genes were down-regulated (green boxes), highlighting those involved in S-adenosyl-L-methionine, polyamines, and phenylpropanoid biosynthesis. Shikimate and folate biosynthesis related genes were also down-regulated. Yellow boxes represent paralogs with opposite transcriptional responses, and whitish-green boxes represent soybean genes not differentially expressed.

A) Read mapping with BWA-MEM.

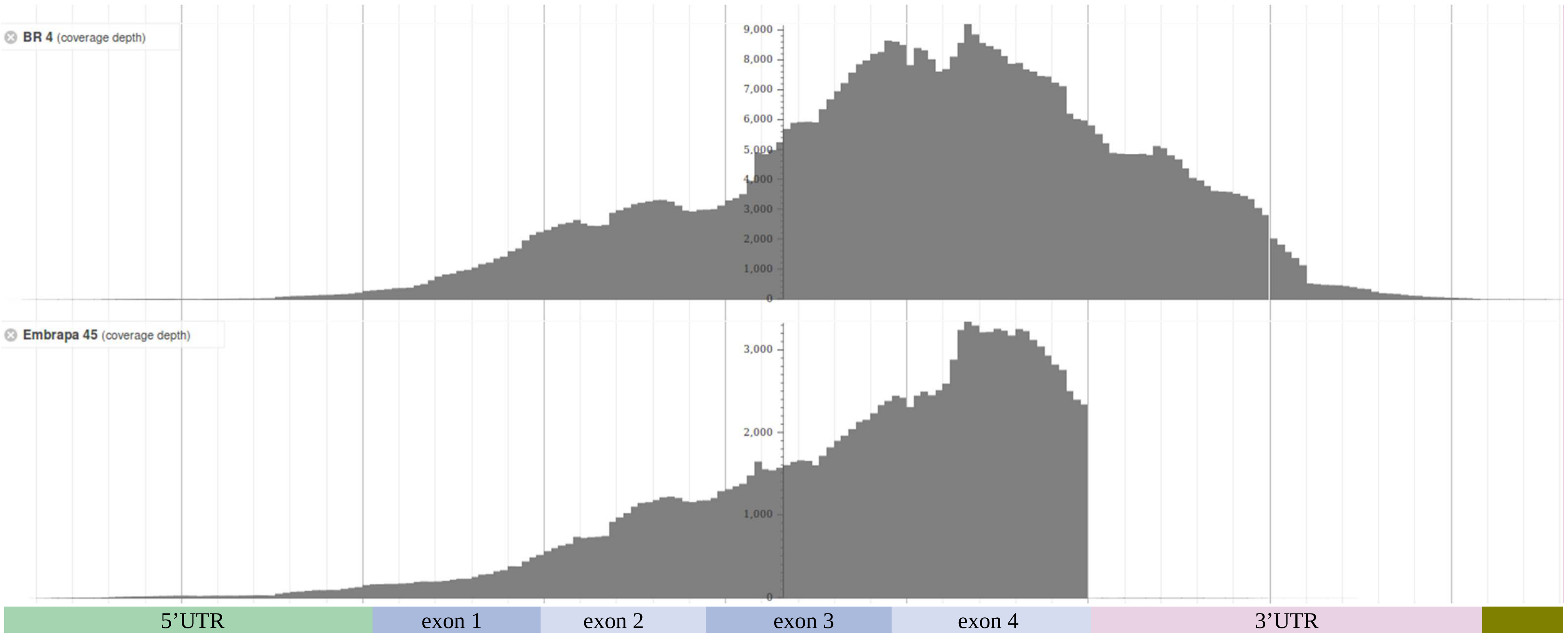

B) *De novo* assembly using Trinity.

BR 4

AAAAAATTACTGTTTGGATATATTCCACTATTTAGAAAGCAAAATGGACTACGAAAACTTGAGTAACAAGGTAAGCCACACAAATGGGAATGA
CTCCCCATTACAATGAAGGGCCAACTTCATTTTCAATGAATCCCACTATAAAAACTTTAGCAATGCAAAAGCTAAAACATCAACCATTTCCTCA
TCCACTTTCACTGGAATCACAATCCTGAAACAAAAACATCTTAGCATTTAACATACTACTAGACAACATGACCACCACATTGGAAAGAGGTTTC
TCGGAAGAGCAAGAAGCTCTGGTGGTGAAGTCATGGAATGTCATGAAGAAGAATTCTGGAGAGTTGGGTCTCAAGTTTTTCTTGAAAATATTT
GAGATTGCTCCATCAGCTCAGAAATTGTTCTCATTCTTGAGAGATTCAACGGTTCCTTTGGAGCAAAATCCCAAGCTCAAGCCCCATGCCGTGT
CTGTCTTTGTAATGACCTGTGATTCAGCAGTTCAGCTGCGGAAGGCCGGGAAAGTCACTGTCAGAGAATCAAACTTGAAAAAATTAGGTGCTA
CCCATTTTAGAACCGGCGTAGCAAACGAGCATTTCGAGGTGACAAAGTTTGCACTGTTGGAGACCATAAAAGAAGCTGTACCAGAAATGTGGT
CACCGGCTATGAAGAATGCATGGGGAGAAGCTTATGATCAGCTGGTCGATGCCATTAAATCTGAAA**TGAAACCACCCTCCTCTTAGACTCC**A
GTTTAAGCAGTTCCTTTCCTTCCCTCTCAATTCTCAAATTGTTATATTAATAAAAGTGAGAAAGTTTAGGCTTGTGCTTTTATTTTGTGTGAATGT
AATATACTTTGTGTACGTAGACTTGGCTATTGGGAGTTGCTA**GGTTGGGAAGTGTTTCGCATTC**AACAATTCTGTAGTTGAAGGTGATTAAAT
GAATTATAGCTATTTGTTTCTTTATACTTATCTGCGATTCAACATAATTCTTCCCGATCACTTATGTGATTGCACCAATATCCTTTGTTGTTTTTAT
GCATAACACACACGTTATGGCAAAAGATAACCCAGTTGGACATGATT

Embrapa 45

AAAAAATTACTGTTTGGATATATTCCACTATTTAGAAAGCAAAATGGACTACGAAAACTTGAGTAACAAGGTAAGCCACACAAATGGGAATGA
CTCCCCATTACAATGAAGGGCCAACTTCATTTTCAATGAATCCCACTATAAAAACTTTAGCAATGCAAAAGCTAAAACATCAACCATTTCCTCA
TCCACTTTCACTGGAATCACAATCCTGAAACAAAAACATCTTAGCATTTAACATACTACTAGACAACATGACCACCACATTGGAAAGAGGTTTC
TCGGAAGAGCAAGAAGCTCTGGTGGTGAAGTCATGGAATGTCATGAAGAAGAATTCTGGAGAGTTGGGTCTCAAGTTTTTCTTGAAAATATTT
GAGATTGCTCCATCAGCTCAGAAATTGTTCTCATTCTTGAGAGATTCAACGGTTCCTTTGGAGCAAAATCCCAAGCTCAAGCCCCATGCCGTGT
CTGTCTTTGTAATGACCTGTGATTCAGCAGTTCAGCTGCGGAAGGCCGGGAAAGTCACTGTCAGAGAATCAAACTTGAAAAAATTAGGTGCTA
CCCATTTTAGAACCGGCGTAGCAAACGAGCATTTCGAGGTGACAAAGTTTGCACTGTTGGAGACCATAAAAGAAGCTGTACCAGAAATGTGGT
CACCGGCTATGAAGAATGCATGGGGAGAAGCTTATGATCAGCTGGTCGATGCCATTAAATCTGAAA**TGAAACCACCCTCCTCTTAGACTCC**A
GTTTAAGCAGTTCCTTTCCTTCCCTCTCAATTCTCAAATTGTTATATTAATAAAAGTGAGAAAGTTTAGGCTTGTGCTTTTATTTTGTGTGAATGT
AATATACTTTGTGTACGTAGACTTGGCTATTGGGAGTTGCTA**GGTTGGGAAGTGTTTCGCATTC**AACAATTCTGTAGTTGAAGGTGATTAAAT
GAATTATAGCTATTTGTTTCTTTATACTTATCTGCGATTCAACATAATTCTTCCCGATCACTTATGTGATTGCACCAATATCCTTTGTTGTTTTTAT
GCATAACACACACGTTATGGCAAAAGATAACCCAGTTGGACATGATT

Fig B. Structural divergence of non-symbiotic hemoglobin Glyma11g12980.1 transcript between BR 4 and Embrapa 45 cultivars by mapping (A) and *de novo* assembly (B) of reads. (A) Glyma11g12980.1 sequence from Phytozome was used as reference for mapping. The total mapped reads for 54,174 predicted protein-coding genes in soybean genome (assembly Glyma 1.1) were 16,845,755 from BR 4 and 19,966,427 from Embrapa 45. Sequence downstream to Phytozome 3'UTR is filled of brown color. (B) Nucleotides not found by *de novo* assembly are represented by red font color. The two bold and underlined sequences are targets of qRT-PCR primer pair (Table D in S1 Supporting Information).

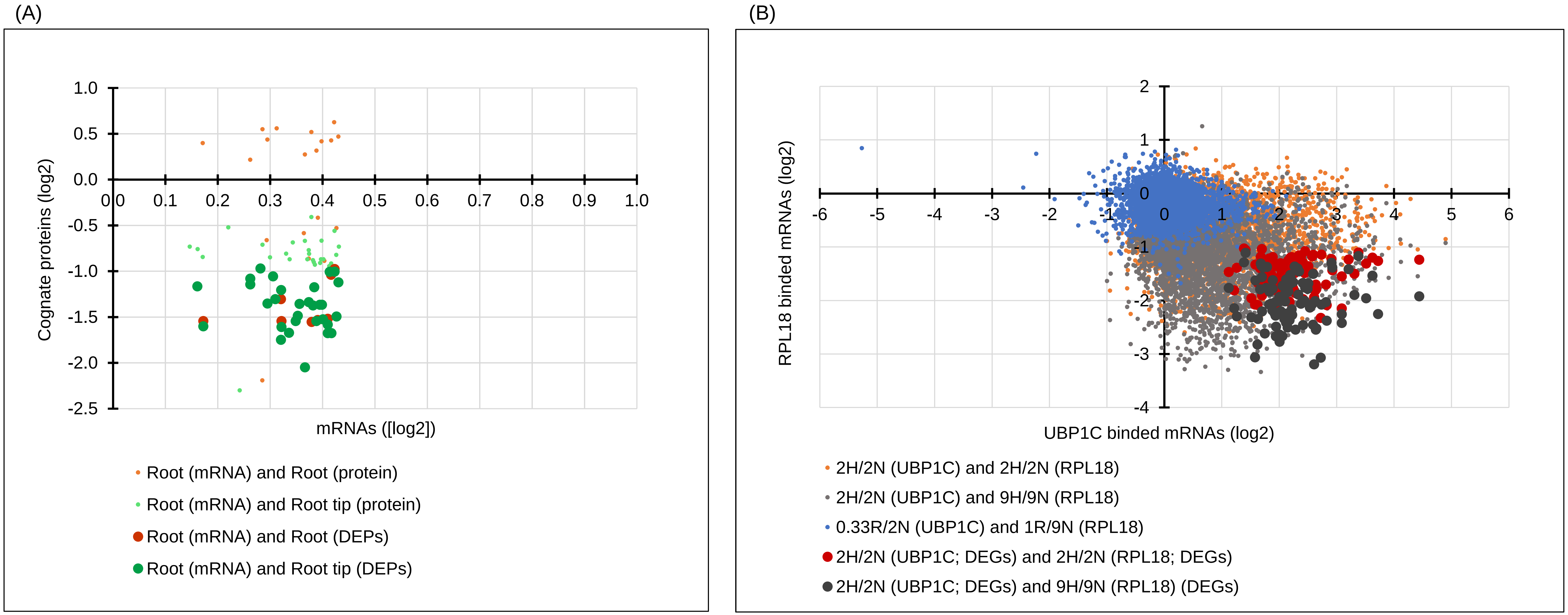

Fig C. Steady-state stable mRNAs tend to be translationally down-regulated (A), decreasing their association with ribosomes (of which include RPL18 component) by their sequestration into stress granules (ribonucleoprotein complexes including UBP1C) under hypoxia, whereas upon reoxygenation they are rapidly released from stress granules forming new polyribosomes (B). (A) The highest absolute fold-change values from our present top 500 steady-state stable mRNAs data (S500) were compared with fold changes from cognate proteins (n = 68) from 2 days flooded soybean plants [79]; differentially expressed proteins (DEPs): log2-transformed fold-change ≥ 1 (up), ≤ -1 (down), $p < 0.01$. (B) Arabidopsis thaliana immunopurified ribosome and stress granule associated mRNA data sets, whose steady-state total mRNAs (n = 4853) changed from 1.35 (log2: 0.43) to -1.35 (log2: -0.43) to hypoxia (also observed in our S500 data), were used from Branco-Price et al. [80] and Sorenson and Bailey-Serres [81] studies, respectively. 2N, 2h non-stress; 2H, 2 h hypoxia-stress; 9N, 9 h non-stress; 9H, 9 h hypoxia stress; 1R, 1 h reoxygenation after 9 h hypoxia-stress; 0.33R, 20 minutes reoxygenation after 2 h hypoxia-stress; DEGs (polyribosomes and stress granules conditions): log2- transformed fold-change ≥ 1 (up), ≤ -1 (down), adj. $p < 0.01$.

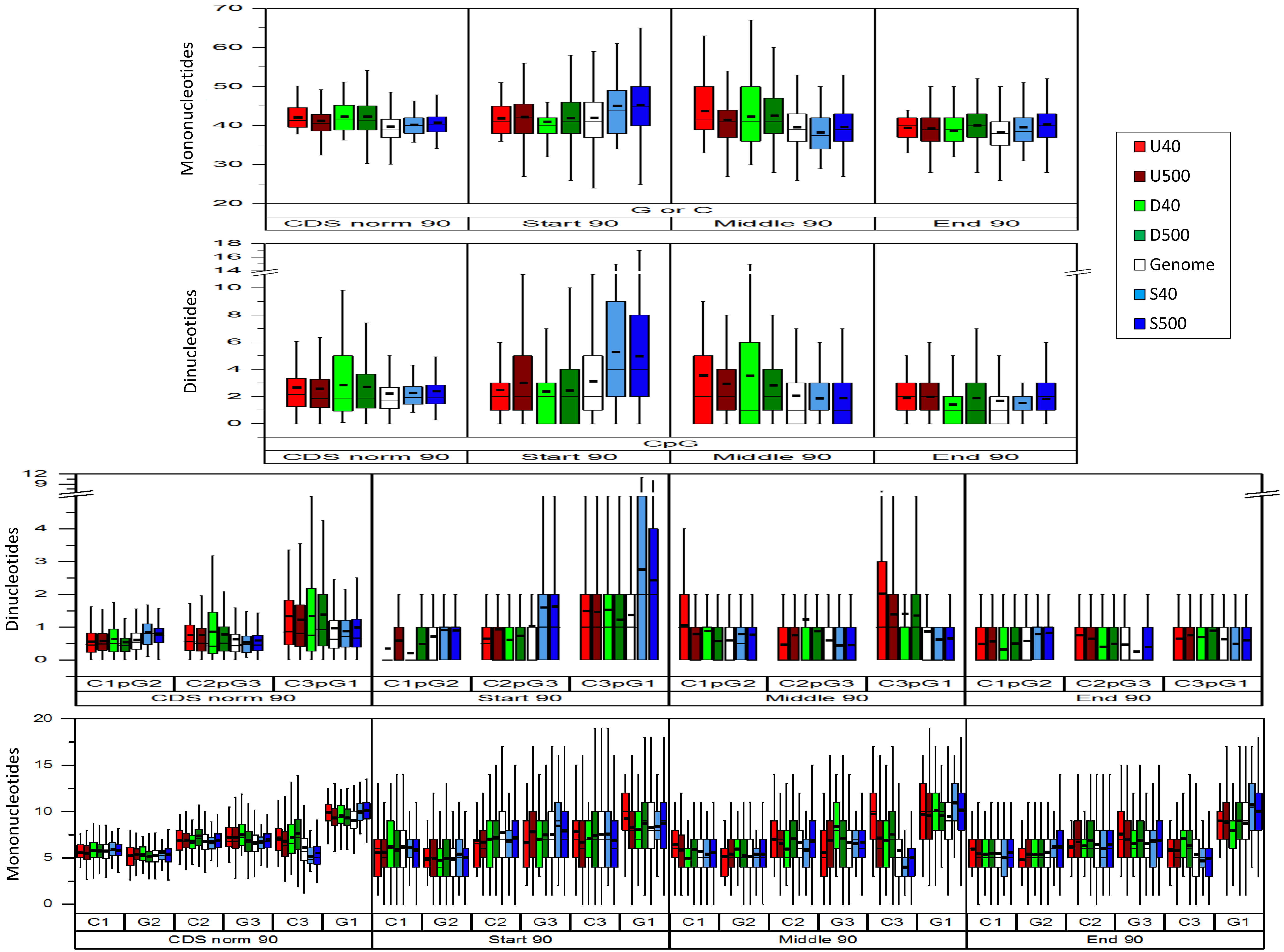

Fig D. Compositional features for full (normalized to 90 nucleotides), start, middle, and end 90 nucleotides length from coding sequences from different soybean gene groups. The groups are formed by top 40 and 500 ranked up- (U), down- (D), and stable-genes (S) to hypoxia, and genome. Phosphate bonds in dinucleotides are represented by “p”, and numbers are nucleotide position in codon. The box is determined by the 25th and 75th percentiles with a line as the median and a black square as the mean of the data. Error bars extend 1.5 times the interquartile range from the 25th and 75th percentiles. Statistical significance from pairwise comparisons are provided in Table C in S1 Supporting Information.

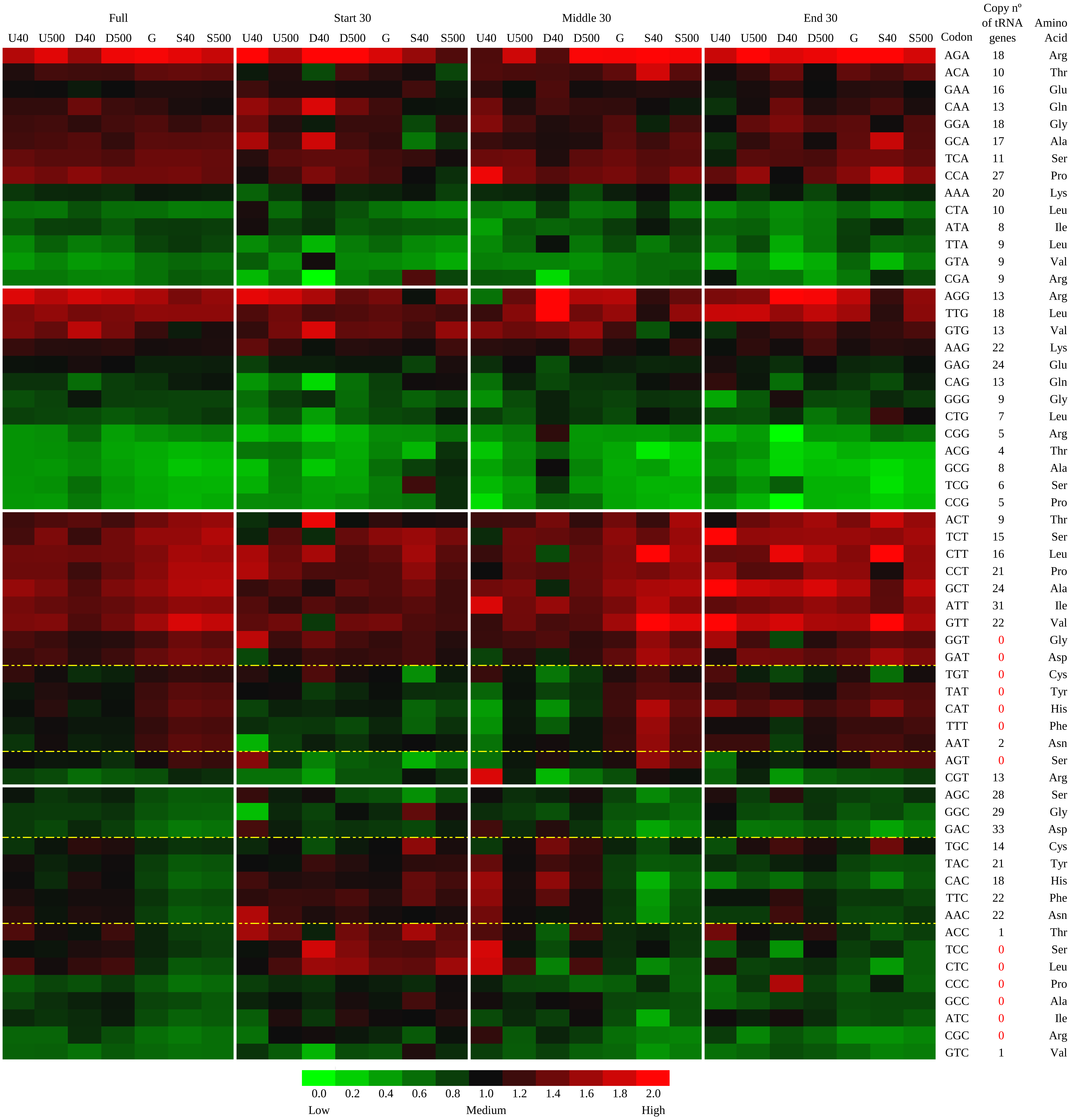

Fig E. Heat map of RSCU (Relative Synonymous Codon Usage) for full, first, middle, and last 30 CDS (coding sequence) codons length from different soybean gene groups. The groups are formed by top 40 and 500 ranked up- (U), down- (D), and stable-genes (S) to hypoxia, and genome (G). Asp, Cys, Tyr, His, Phe, and Asn are coding by 2-fold degenerate pyrimidine ending codons.

References

79. Wang X, Oh M, Sakata K, Komatsu S. Gel-free/label-free proteomic analysis of root tip of soybean over time under flooding and drought stresses. J Proteomics. Elsevier B.V.; 2016;130: 42–55. doi:10.1016/j.jprot.2015.09.007

80. Branco-Price C, Kaiser KA, Jang CJH, Larive CK, Bailey-Serres J. Selective mRNA translation coordinates energetic and metabolic adjustments to cellular oxygen deprivation and reoxygenation in Arabidopsis thaliana. Plant J. 2008;56: 743–755. doi:10.1111/j.1365-313X.2008.03642.x

81. Sorenson R, Bailey-Serres J. Selective mRNA sequestration by OLIGOURIDYLATE-BINDING PROTEIN 1 contributes to translational control during hypoxia in Arabidopsis. Proc Natl Acad Sci. 2014;111: 2373–2378. doi:10.1073/pnas.1314851111

100. Jian B, Liu B, Bi Y, Hou W, Wu C, Han T. Validation of internal control for gene expression study in soybean by quantitative real-time PCR. BMC Mol Biol. 2008;14: 1–14. doi:10.1186/1471-2199-9-59

101. Byfield GE, Xue H, Upchurch RG. Two Genes from Soybean Encoding Soluble Δ9 Stearoyl-ACP Desaturases. Crop Sci. 2006;46: 840. doi:10.2135/cropsci2005.06-0172
